# Sex differences in risk factors for Alzheimer dementia encephalopathy patients

**DOI:** 10.3389/frdem.2025.1593788

**Published:** 2025-05-26

**Authors:** Connor John O’Brien, James Wayne Patterson, Dami Taiwo Ojo, Nathan Gerhard Faulstich, Killian Joseph Bucci, Philip Cole Brewer, Adebobola Imeh-Nathaniel, Emmanuel I. Nathaniel, Laurie Roley, Richard Goodwin, Thomas I. Nathaniel

**Affiliations:** ^1^School of Medicine Greenville, University of South Carolina, Greenville, SC, United States; ^2^Department of Biology, North Greenville University, Tigerville, SC, United States; ^3^PRISMA Health, Greer, SC, United States; ^4^Department of Biomedical Engineering, University of South Carolina, Columbia, SC, United States

**Keywords:** Alzheimer’s dementia, encephalopathy, risk factors, sex, male and female patients

## Abstract

**Background:**

The objective is to identify risk factors that contribute to sex differences in Alzheimer dementia (AD) patients with encephalopathy (ADEN) and determine whether these factors are different between male and female ADEN patients. This is the first large-scale study comparing sex-specific ADEN risk profiles.

**Methods:**

Our retrospective cohort study analyzed data collected from February 2016 to August 2020. It included a total of 128,769 AD patients, among whom 41,266 AD patients also presented with encephalopathy, compared to 87,503 AD patients that did not. The univariate analysis was used to determine differences in risk factors for male and female AD patients. Multivariate analysis predicted specific risk factors associated with male and female ADEN patients.

**Result:**

In the adjusted analysis, males presented with hypertension (OR = 1.144, 95% CI, 1.094–1.197, *p* < 0.001), peripheral vascular disease (OR = 1.606, 95% CI, 1.485–1.737, *p* < 0.001), atrial fibrillation (OR = 1.555, 95% CI, 1.443–1.676, *p* < 0.001), hallucinations (OR = 1.406, 95% CI, 1.119–1.766, *p* = 0.003), and traumatic head injury (OR = 3.211, 95% CI, 2.346–4.395, *p* < 0.001). Females presented with osteoporosis (OR = 0.307, 95% CI, 0.278–0.340, *p* < 0.001), unspecified cancer (OR = 0.615, 95% CI, 0.512–0.740, *p* < 0.001), anxiety (OR = 0.609, 95% CI, 0.565–0.655, *p* < 0.001), urinary tract infections (UTI) (OR = 0.451, 95% CI, 0.423–0.481, *p* < 0.001), upper respiratory infections (URI) (OR = 0.531, 95% CI, 0.432–0.653, *p* < 0.001) and gastrointestinal ulceration (OR = 0.338, 95% CI, 0.269–0.424, *p* < 0.001).

**Conclusion:**

Our analysis identified risk factors that contribute to sex differences in ADEN. This difference was fully mediated by peripheral vascular disease, atrial fibrillation, hallucinations, and traumatic head injury for males and unspecified cancer, anxiety, urinary tract infections, upper respiratory infections, and gastrointestinal ulceration for females. These findings provide valuable insights into the risk factors that can be managed to improve the care of male and female ADEN patients.

## Introduction

Alzheimer’s disease (AD) is a progressive neurodegenerative disorder that leads to the death of nerve cells in the brain, resulting in memory loss and cognitive decline (Targa Dias Anastacio et al., 2022). The development of AD is associated with the accumulation of amyloid-beta (Aβ) plaques and hyperphosphorylated tau proteins (Rajmohan and Reddy, 2017). These abnormalities disrupt communication between pre- and post-synaptic neurons and obstruct the transportation of essential nutrients and molecules, ultimately causing neuronal death. AD’s primary symptoms include memory, language, and thinking skills (Cerejeira et al., 2012). In addition, patients may experience personality, mood, and behavior changes, and as the disease progresses, it increasingly impacts other cognitive functions (Alzheimer's Association Report, 2024).

Encephalopathy can occur in individuals with dementia, particularly when they experience metabolic or toxic stress (Krishnan et al., 2014). Additionally, it can be a cause of dementia, such as in cases of chronic traumatic encephalopathy (Gavett et al., 2011). A patient presenting with encephalopathy and dementia experiences cognitive decline and altered mental status. Symptoms often include memory loss, confusion, and difficulty with reasoning alongside broader signs of encephalopathy, such as reduced awareness, fluctuating attention, and changes in behavior (Hermann and Zerr, 2022). These symptoms are typically the result of systemic issues, including metabolic disturbances, intoxication, or severe infections.

While dementia generally refers to a progressive neurodegenerative disease, encephalopathy with dementia can be triggered by various factors. For instance, metabolic issues such as the liver not being able to remove toxins from the bloodstream and hypoglycemia can cause acute encephalopathy, which may progress to dementia if left untreated (Marois et al., 2019). In addition, exposure to some toxic materials, including heavy metals, can cause encephalopathy, which may progressively develop into dementia over time (Bakulski et al., 2020). Chronic traumatic encephalopathy can also begin with symptoms of encephalopathy that gradually worsen into dementia (Lakhan and Kirchgessner, 2012). Furthermore, encephalitis can cause acute encephalopathy, resulting in long-term cognitive impairment that resembles dementia (Bastiaansen et al., 2021; Miller et al., 2022). Alzheimer’s disease and encephalopathy share some neuropathological features, such as the presence of neurofibrillary tangles and hyperphosphorylated tau (Singh et al., 2025; Stanley et al., 2023). However, they are generally considered distinct entities, with encephalopathy resulting from repeated TBI and AD having a different etiology, including a combination of genetic, lifestyle, and environmental factors that affect the brain over time (Izzy et al., 2022; Agbomi et al., 2022). Encephalopathy and Alzheimer’s dementia disease can coexist in the same individuals. This can lead to a more complex clinical presentation and potentially a more severe course of the disease, potentially overlapping neuropathologically, resulting in a more rapid progression of the AD and a more severe clinical course compared to having only AD.

In terms of sex differences, males typically show more severe symptoms and developmental delays when affected by X-linked encephalopathies (Mignot et al., 2019). Since males have only one X chromosome, X-linked mutations have a greater impact. However, females can also experience symptoms of encephalopathy with variations in severity depending on the patterns of X inactivation (Migeon, 2020). AD tends to be more rapid among elderly females than males. Females are often diagnosed earlier in the course of AD than their male counterparts, which can complicate the assessment of post-diagnosis longevity (Podcasy and Epperson, 2016). Nonetheless, data focusing on the mortality of AD and all-cause dementia indicate that males generally have a shorter lifespan, regardless of age at diagnosis (Hale et al., 2020).

Risk factors for AD patients with a combined encephalopathy (ADEN) include advanced age, head trauma, exposure to toxins including drugs, chemicals, radiation, severe infections, and organ failure such as liver and kidney (Frontera et al., 2021). In addition, electrolyte imbalances, metabolic disorders, multiple medications, substance abuse, nutritional deficiencies, severe medical conditions affecting multiple body systems, and a family history of neurodegenerative disease have also been reported as risk factors for ADEN (Arnold et al., 2016). Other contributing factors to neurologic dysfunction ADEN are the use of multiple neurotropic drugs, as well as major surgery, organ failure, electrolyte disturbance, and endocrine disease (Chaudhry and Duggal, 2014).

Smoking, coronary artery disease, and brain injury with loss of consciousness are more common among male than female AD patients (Durazzo et al., 2014; Broughton et al., 2025). However, other risk factors, such as diabetes, obesity, and hypertension, are also more common among males, but females are disproportionately at risk for AD when these conditions are present (Mielke et al., 2022; Gainey et al., 2024). The longer life spans observed in females do not fully explain the sex bias for AD but increase the overall prevalence of all-cause AD in females among the oldest of the old (Lopez-Lee et al., 2024). In addition, females are at greater risk of developing AD, whereas males are at greater risk of developing vascular dementia (Beam et al., 2018; Brewer et al., 2024).

Sex differences in disease prevalence and biological mechanisms are significant and widespread, affecting everything from basic physiological functions to disease susceptibility and response to treatment (Chlamydas et al., 2022; Imeh-Nathaniel et al., 2024). A complex interplay of genetic, hormonal, and epigenetic factors influences these differences. Moreover, there are sex-specific diseases, such that some diseases are almost exclusively found in one sex. For example, cancer in women and prostate cancer in men are both significant reproductive system cancers, and they affect different organs and have different incidence and risk factors (Jianhui et al., 2023). Many conditions, including autoimmune diseases, are more common in women, while others, like autism, are more common in men (Becker, 2012). Gonadal hormones like estrogen and testosterone are crucial biological mechanisms that significantly impact various physiological processes and influence disease susceptibility in both men and women (Boese et al., 2017).

In general, hormones are vital chemical messengers that influence various bodily functions, impacting sexual development, reproduction, bone health, cardiovascular health, and even immune responses in both men and women in disease conditions (Sciarra et al., 2023). For example, females are reported to present a higher risk of developing encephalopathy and dementia compared to males, with key differences in such factors as hormone fluctuations during post-menopause, cardiovascular health, and depression (Bleiler and Thies, 2013; Suter et al., 2023; Ayus et al., 1992; Dilwali et al., 2021). However, the specific risk factors that contribute to sex differences in patients who present a combined encephalopathy and dementia are yet to be fully understood. Females are more likely to develop dementia, especially AD, compared to males (Beam et al., 2018), and males tend to exhibit more severe symptoms of encephalopathy compared to females (Tenembaum, 2013; Coker-Ayo et al., 2022), indicating that risk factors in males and females with ADEN may not be the same. This study evaluates the differences in risk factors between male and female encephalopathy in dementia patients. Specifically, our objectives are to (A) analyze the differences in risk factors for male and female ADEN patients and (B) investigate whether these differences can account for the sex disparities observed in ADEN patients. By understanding these sex-related risk factors, we can provide important insights to other healthcare professionals, ultimately enhancing clinical practice in the care of male and female patients with combined encephalopathy and dementia.

## Methods

### Study population

This study analyzed patients treated at Prisma Health-Upstate (previously Greenville Health System) diagnosed with dementia from February 2016 to August 2020. The Prisma Health Institutional Review Board (IRB) approved the study design. Inclusion criteria included patients with confirmed cases of ADEN only and AD patients without encephalopathy. The non-encephalopathy cohort of dementia patients included etiologies such as vascular dementia, Alzheimer’s disease, mixed dementia, frontotemporal dementia, Lewy body dementia, Parkinson’s disease, and normal pressure hydrocephalus. The cohort with encephalopathy included patients with both acute and chronic encephalopathy, including metabolic encephalopathy, toxic encephalopathy, uremic encephalopathy, HIV encephalopathy, chronic traumatic encephalopathy, infectious encephalopathy, septic encephalopathy, Hashimoto encephalopathy, Glycine encephalopathy, Leukoencephalopathy, Static encephalopathy, and Progressive encephalopathy.

We extracted data for demographics, medical history, medication use, social risk factors, and lab values at admission. The demographic variables included age, race, sex, and ethnicity. Race was categorized as White, Black, or Other. Ethnicity was defined as a binary variable of Hispanic/Latino or Non-Hispanic/Non-Latino. Medication use was grouped by drug class when applicable. Cholinesterase inhibitors (ChEIs included donepezil, galantamine, and rivastigmine). Second-generation antipsychotics (SGAs) included aripiprazole, olanzapine, and risperidone, and selective serotonin reuptake inhibitors (SSRIs) included citalopram, escitalopram, and paroxetine. The social risk factors analyzed were alcohol and tobacco use. A positive value for the alcohol variable denotes patients with either current and/or prior consumption, and similar criteria were used for tobacco use. Lab values included data for serum folate (B9), cobalamin (B12), vitamin D, Thyroid Stimulating Hormone (TSH), magnesium, calcium, and homocysteine.

### Statistical analysis

All statistical analyses were conducted using the Statistical Package for Social Sciences version 29.0 for Mac (SPSS, Chicago, IL). Univariate analysis was used to evaluate differences in risk factors between AD patients with encephalopathy (ADEN) and those without. Discrete variables were analyzed using the Pearson χ2 test, while continuous variables were examined with a Student *t*-test. Variables that were statistically significant or approaching significance (*p*-value < 0.30) were utilized to construct a multivariable logistic regression model. We used a liberal *p*-value threshold (*p* < 0.30) for variable inclusion in our multivariable modeling because it allows the consideration of a wider range of potentially relevant variables in the final model, especially since our preliminary analysis suggests they might significantly impact the outcome. This approach prioritizes the inclusion of variables of potential clinical relevance, even if their statistical significance is not strong in a purely statistical sense. Each model was developed using backward logistic regression by systematically eliminating less significant variables until an optimal model was achieved. We used the backward approach because it provides the advantage of assessing the joint predictive power of variables in our model by starting with a full model and iteratively removing non-significant ones. It also helped to retain important confounding variables that allow us to develop richer models. We validated our model’s predictive accuracy by comparing model predictions with theoretical expectations, using new data to validate predictions, and analyzing residuals to assess model fit. In our regression analysis, we controlled confounding factors, including age and comorbidities, which were adjusted for by including them as independent variables in the regression model. This approach allows our regression model to estimate the unique contribution of each variable to the outcome while controlling for the influence of other variables. Odds ratios (OR) with a 95% confidence interval for each variable in the final model were used to identify the factors associated with AD patients with encephalopathy compared to those without. The same method was applied to assess risk factors associated with males versus females stratified by AD patients with and without encephalopathy. The sensitivity and specificity of the model were evaluated using the overall classification percentage and the area under the Receiver Operating Characteristic Curve (AUROC). Additionally, the Hosmer-Lemeshow test and correlation matrix were employed to assess multicollinearity among variables in the model. In cases where two variables exhibited a strong association (r > 0.7), the variable with the lower OR was removed, and the model was re-run. A *p*-value of less than 0.05 was considered significant for all statistical tests.

## Results

### Demographic characteristics and risk factors for male and female patients

A total of 128,769 patients with AD patients were identified, among whom 41,266 were ADEN compared to 87,503 AD patients without encephalopathy (see Table 1). ADEN patients were predominantly male African Americans (*p* < 0.001). Additionally, these patients were more likely to present with a history of peripheral vascular disease (PVD), atrial fibrillation, urinary tract infections (UTIs), insulin use, gastrointestinal (GI) ulcers, congestive heart failure (CHF), chronic obstructive pulmonary disease (COPD), vasospasm, pneumonia, rheumatoid arthritis, subclavian artery disease, and gastric erosion (*p* < 0.001). Furthermore, they were more likely to report a history of small cell lung carcinoma (*p* < 0.001), squamous cell lung carcinoma (*p* < 0.001), large cell lung carcinoma (*p* = 0.012), and unspecified cancer (*p* < 0.001). A notable increase in the incidence of cutaneous ulcers (*p* = 0.041) and tobacco use (*p* < 0.001) was also observed among these patients.

**Table 1:** Demographic and clinical characteristics of AD patients stratified by absence or presence of encephalopathy.

Characteristic	Dementia without encephalopathy	Dementia with encephalopathy	*p-*value
Number of patients	87,503	41,266	
Male gender No. (%)	36,671 (41.9)	19,202 (46.5)	<0.001^*^^a^
Age group: No. (%)			<0.001^*^^a^
<50	15,287 (17.5)	6,174 (15.0)	
50–59	7,165 (8.2)	7,147 (17.3)	
60–69	11,770 (13.5)	10,143 (24.6)	
70–79	21,101 (24.1)	9,045 (21.9)	
> = 80	32,180 (36.8)	8,757 (21.2)	
Mean ± SD	69.38 ± 21.877	66.93 ± 16.226	<0.001^*^^b^
Race: No (%)			<0.001^*^^a^
White	70,893 (81.0)	31,809 (77.1)	
Black	12,618 (14.4)	8,156 (19.8)	
Other	3,992 (4.6)	1,301 (3.1)	
Hispanic ethnicity: No. (%)	1952 (2.2)	628 (1.5)	<0.001^*^^a^
Medical history: No. (%)			
Insomnia	6,783 (7.8)	2,123 (5.1)	<0.001^*^^a^
Hypertension	46,690 (53.4)	21,346 (51.7)	<0.001^*^^a^
Dyslipidemia	23,560 (26.9)	9,039 (21.9)	<0.001^*^^a^
Cerebrovascular accident	9,593 (11.0)	3,928 (9.5)	<0.001^*^^a^
Osteoporosis	9,352 (10.7)	2,832 (6.9)	<0.001^*^^a^
Gait dysfunction	3,650 (4.2)	867 (2.1)	<0.001^*^^a^
Peripheral vascular disease	5,174 (5.9)	3,481 (8.4)	<0.001^*^^a^
Atrial fibrillation	6,720 (7.7)	3,718 (9.0)	<0.001^*^^a^
Hallucination	858 (1.0)	359 (0.9)	0.056^a^
Cancer (unspecified)	722 (0.8)	591 (1.4)	<0.001^*^^a^
Anxiety	9,531 (10.9)	4,255 (10.3)	0.002^*^^a^
Urinary tract infection	8,767 (10.0)	5,922 (14.4)	<0.001^*^^a^
Upper respiratory infection	2,204 (2.5)	601 (1.5)	<0.001^*^^a^
Thyroid disease (Unspecified)	545 (0.6)	138 (0.3)	<0.001^*^^a^
Insulin use	633 (0.7)	534 (1.3)	<0.001^*^^a^
Secondary dementia	2,153 (2.5)	253 (0.6)	<0.001^*^^a^
Cold sore	292 (0.3)	34 (0.1)	<0.001^*^^a^
Frailty	145 (0.2)	33 (0.1)	<0.001^*^^a^
Gastrointestinal ulceration	532 (0.6)	418 (1.0)	<0.001^*^^a^
Lung adenocarcinoma	152 (0.2)	58 (0.1)	0.169^a^
Small cell carcinoma of lung	89 (0.1)	112 (0.3)	<0.001^*^^a^
Squamous cell carcinoma of lung	42 (0.0)	54 (0.1)	<0.001^*^^a^
Large cell carcinoma of lung	0 (0.0)	3 (0.0)	0.012^*^^a^
Mild cognitive impairment	6,366 (7.3)	385 (0.9)	<0.001^*^^a^
Headache (unspecified)	1757 (2.0)	577 (1.4)	<0.001^*^^a^
Congestive heart failure	4,746 (5.4)	3,898 (9.4)	<0.001^*^^a^
Obstructive sleep apnea	9,408 (10.8)	3,875 (9.4)	<0.001^*^^a^
Arteriosclerosis	222 (0.3)	119 (0.3)	0.259^a^
Cutaneous ulcer	149 (0.2)	92 (0.2)	0.041^*^^a^
Psychosis	761 (0.9)	229 (0.6)	<0.001^*^^a^
Chronic obstructive pulmonary disease	7,998 (9.1)	5,788 (14.0)	<0.001^*^^a^
Traumatic head injury	509 (0.6)	220 (0.5)	0.278^a^
Vasospasm	0 (0.0)	9 (0.0)	<0.001^*^^a^
Pneumonia	8,134 (9.3)	8,056 (19.5)	<0.001^*^^a^
Rheumatoid arthritis	1,018 (1.2)	676 (1.6)	<0.001^*^^a^
Ductal carcinoma of breast	4 (0.0)	3 (0.0)	0.540^a^
Subclavian artery disease	0 (0.0)	15 (0.0)	<0.001^*^^a^
Teratoma	5 (0.0)	0 (0.0)	0.125^a^
Microangiopathy	2 (0.0)	0 (0.0)	0.331^a^
Down syndrome	140 (0.2)	13 (0.0)	<0.001^*^^a^
Gastric erosion	3 (0.0)	10 (0.0)	<0.001^*^^a^
Senile tremor	10 (0.0)	0 (0.0)	0.030^*^^a^
Lab values: Mean ± SD			
Folate (B9)	11.924 ± 4.0659	11.367 ± 4.2398	<0.001^*^^b^
Cobalamin (B12)	516.50 ± 188.798	827.00 ± 485.412	0.452^b^
Vitamin D	29.740 ± 15.1571	27.496 ± 16.0328	0.002^*^^b^
TSH	3.02940 ± 9.817910	3.55786 ± 11.584016	0.001^*^^b^
Magnesium	1.960 ± 0.3254	1.932 ± 0.3485	<0.001^*^^b^
Serum calcium	9.134 ± 0.6654	8.983 ± 0.7555	<0.001^*^^b^
Homocysteine	14.732 ± 15.1210	14.591 ± 8.1239	0.941^b^
Alcohol use (%)	27.9	28.7	0.004^*^^b^
Tobacco use (%)	49.3	65.8	<0.001^*^^a^
Medications No. (%)			
Central acetylcholinesterase inhibitor	26,642 (30.4)	3,475 (8.4)	<0.001^*^^a^
Second Generation Antipsychotic	13,995 (16.0)	6,641 (16.1)	0.650^a^
Selective Serotonin Reuptake Inhibitor	28,829 (32.9)	11,992 (29.1)	<0.001^*^^a^
Memantine	17,459 (20.0)	2,324 (5.6)	<0.001^*^^a^
Buspirone	8,137 (9.3)	4,040 (9.8)	0.005^*^^a^
Valproate	13,816 (15.8)	5,723 (13.9)	<0.001^*^^a^

a Pearson’s Chi-Squared test.

b Student’s *T* test.

^*^ *p*-value <0.05.

AD patients without encephalopathy tended to be older, predominantly Caucasian, and of Hispanic ethnicity (*p* < 0.001). They were also more likely to present with a range of medical histories, including insomnia, hypertension (HTN), developmental language disorder (DLD), cerebrovascular accidents (CVA), osteoporosis, gait dysfunction, upper respiratory infections (URI), thyroid disease, secondary dementia, cold sores, frailty, mild cognitive impairment, unspecified headaches, obstructive sleep apnea (OSA), psychosis, and Down syndrome (*p* < 0.001). Additionally, these patients showed a higher occurrence of anxiety (*p* = 0.002) and senile tremor (*p* = 0.030). In contrast, ADEN patients were more likely to present with elevated serum levels of TSH (*p* = 0.001). Conversely, those without encephalopathy were more likely to have higher levels of folate, magnesium, calcium (*p* < 0.001), and vitamin D (*p* = 0.002). ADEN patients were more likely to be prescribed buspirone (*p* = 0.005), whereas AD patients without encephalopathy were more frequently treated with ChEIs and SSRIs, including memantine and valproate (*p* < 0.001).

The demographic and risk factors of AD patients, both with and without encephalopathy, stratified by sex, are presented in Table 2. In the cohort of ADEN patients, males were significantly more likely to be of Hispanic ethnicity and to have a history of alcohol and tobacco use (*p* < 0.001). They were also more likely to have a medical history that included PVD, atrial fibrillation, traumatic head injury, and subclavian artery disease (*p* < 0.001), as well as symptoms such as hallucinations (*p* = 0.004) and psychosis (*p* = 0.007). Additionally, males exhibited a higher serum magnesium level compared to females (*p* < 0.001).

**Table 2:** Demographic and clinical characteristics of male and female AD patients stratified by the presence or absence of encephalopathy.

Characteristic	No encephalopathy		*p*-value	Encephalopathy		
	Male	Female		Male	Female	*p*-value
Number of patients	36,671	50,832		19,202	22,064	
Age Group: No. (%)			<0.001^*^^a^			<0.001^*^^a^
<50 years	6,637 (18.1)	8,133 (16.0)		2,759 (14.4)	2,789 (12.6)	
50–59	2,706 (7.4)	3,956 (7.8)		3,354 (17.5)	3,587 (16.3)	
60–69	5,323 (14.5)	6,033 (11.9)		4,789 (24.9)	5,019 (22.7)	
70–79	8,816 (24.0)	11,047 (21.7)		4,342 (22.6)	5,156 (23.4)	
80–89	9,380 (25.6)	13,315 (26.2)		2,818 (14.7)	3,414 (15.5)	
> = 90	3,809 (10.4)	8,348 (16.4)		1,140 (5.9)	2099 (9.5)	
Age Mean ± SD	67.44 ± 22.631	70.78 ± 21.206	<0.001^*^^b^	65.78 ± 16.109	67.93 ± 16.261	<0.001*^b^
Race: No (%)			<0.001^*^^a^			<0.001^*^^a^
White	30,147 (82.2)	40,746 (80.2)		14,354 (74.8)	17,455 (79.1)	
Black	4,783 (13.0)	7,835 (15.4)		4,140 (21.6)	4,016 (18.2)	
Other	1741 (4.7)	2,251 (4.4)		708 (3.7)	593 (2.7)	
Hispanic ethnicity: No. (%)	856 (2.3)	1,096 (2.2)	0.078^a^	363 (1.9)	265 (1.2)	<0.001^*^^a^
Medical history: No. (%)						
Alcohol use (%)	34.6	23.1	<0.001^*^^a^	37.6	20.9	<0.001^*^^a^
Tobacco use (%)	62.3	40.0	<0.001^*^^a^	76.5	56.5	<0.001^*^^a^
Insomnia	2,497 (6.8)	4,286 (8.4)	<0.001^*^^a^	913 (4.8)	1,210 (5.5)	<0.001^*^^a^
Hypertension	19,109 (52.1)	27,581 (54.3)	<0.001^*^^a^	9,680 (50.4)	11,666 (52.9)	<0.001^*^^a^
Dyslipidemia	10,406 (28.4)	13,154 (25.9)	<0.001^*^^a^	4,014 (20.9)	5,025 (22.8)	<0.001^*^^a^
Cerebrovascular accident	4,093 (11.2)	5,500 (10.8)	0.111^a^	1800 (9.4)	2,128 (9.6)	0.350^a^
Osteoporosis	1,614 (4.4)	7,738 (15.2)	<0.001^*^^a^	567 (3.0)	2,265 (10.3)	<0.001^*^^a^
Gait dysfunction	1,600 (4.4)	2050 (4.0)	0.016^*^^a^	365 (1.9)	502 (2.3)	0.008^*^^a^
Peripheral vascular disease	2,754 (7.5)	2,420 (4.8)	<0.001^*^^a^	1957 (10.2)	1,524 (6.9)	<0.001^*^^a^
Atrial fibrillation	2,894 (7.9)	3,826 (7.5)	0.045^*^^a^	1918 (10.0)	1800 (8.2)	<0.001^*^^a^
Hallucination	325 (0.9)	533 (1.0)	0.016^*^^a^	194 (1.0)	165 (0.7)	0.004^*^^a^
Cancer (unspecified)	398 (1.1)	324 (0.6)	<0.001^*^^a^	224 (1.2)	367 (1.7)	<0.001^*^^a^
Anxiety	2,940 (8.0)	6,591 (13.0)	<0.001^*^^a^	1,392 (7.2)	2,863 (13.0)	<0.001^*^^a^
Urinary tract infection	2,235 (6.1)	6,532 (12.9)	<0.001^*^^a^	1,651 (8.6)	4,271 (19.4)	<0.001^*^^a^
Upper respiratory infection	651 (1.8)	1,553 (3.1)	<0.001^*^^a^	142 (0.7)	459 (2.1)	<0.001^*^^a^
Thyroid disease (unspecified)	149 (0.4)	396 (0.8)	<0.001^*^^a^	65 (0.3)	73 (0.3)	0.893^a^
Insulin use	201 (0.5)	432 (0.8)	<0.001^*^^a^	164 (0.9)	370 (1.7)	<0.001^*^^a^
Secondary dementia	821 (2.2)	1,332 (2.6)	<0.001^*^^a^	93 (0.5)	160 (0.7)	0.002^*^^a^
Cold sore	56 (0.2)	236 (0.5)	<0.001^*^^a^	13 (0.1)	21 (0.1)	0.332^a^
Frailty	74 (0.2)	71 (0.1)	0.026^*^^a^	7 (0.0)	26 (0.1)	0.004^*^^a^
Gastrointestinal ulceration	63 (0.2)	469 (0.9)	<0.001^*^^a^	108 (0.6)	310 (1.4)	<0.001^*^^a^
Lung adenocarcinoma	44 (0.1)	108 (0.2)	0.001^*^^a^	29 (0.2)	29 (0.1)	0.596^a^
Small cell carcinoma of lung	16 (0.0)	73 (0.1)	<0.001^*^^a^	42 (0.2)	70 (0.3)	0.055^a^
Squamous cell carcinoma of lung	37 (0.1)	5 (0.0)	<0.001^*^^a^	2 (0.0)	52 (0.2)	<0.001^*^^a^
Large cell carcinoma of lung	0 (0.0)	0 (0.0)	N/A	3 (0.0)	0 (0.0)	0.063^a^
Mild cognitive impairment	2,768 (7.5)	3,598 (7.1)	0.008^*^^a^	178 (0.9)	207 (0.9)	0.906^a^
Headache (unspecified)	215 (0.6)	1,542 (3.0)	<0.001^*^^a^	154 (0.8)	423 (1.9)	<0.001^*^^a^
Congestive heart failure	1813 (4.9)	2,933 (5.8)	<0.001^*^^a^	1,594 (8.3)	2,304 (10.4)	<0.001^*^^a^
Obstructive Sleep Apnea	4,652 (12.7)	4,756 (9.4)	<0.001^*^^a^	1,536 (8.0)	2,339 (10.6)	<0.001^*^^a^
Arteriosclerosis	100 (0.3)	122 (0.2)	0.343^a^	53 (0.3)	66 (0.3)	0.662^a^
Cutaneous ulcer	112 (0.3)	37 (0.1)	<0.001^*^^a^	9 (0.0)	83 (0.4)	<0.001^*^^a^
Psychosis	365 (1.0)	396 (0.8)	<0.001^*^^a^	127 (0.7)	102 (0.5)	0.007^*^^a^
Chronic obstructive pulmonary disease	3,405 (9.3)	4,593 (9.0)	0.206^a^	2,476 (12.9)	3,312 (15.0)	<0.001^*^^a^
Traumatic head injury	220 (0.6)	289 (0.6)	0.547^a^	142 (0.7)	78 (0.4)	<0.001^*^^a^
Vasospasm	0 (0.0)	0 (0.0)	N/A	0 (0.0)	9 (0.0)	0.005^*^^a^
Pneumonia	3,616 (9.9)	4,518 (8.9)	<0.001^*^^a^	3,770 (19.6)	4,286 (19.4)	0.595^a^
Rheumatoid arthritis	215 (0.6)	803 (1.6)	<0.001^*^^a^	147 (0.8)	529 (2.4)	<0.001^*^^a^
Ductal carcinoma of breast	0 (0.0)	4 (0.0)	0.089^a^	0 (0.0)	3 (0.0)	0.106^a^
Subclavian artery disease	0 (0.0)	0 (0.0)	N/A	15 (0.1)	0 (0.0)	<0.001^*^^a^
Teratoma	0 (0.0)	5 (0.0)	0.058^a^	0 (0.0)	0 (0.0)	N/A
Microangiopathy	0 (0.0)	2 (0.0)	0.230^a^	0 (0.0)	0 (0.0)	N/A
Down syndrome	91 (0.2)	49 (0.1)	<0.001^*^^a^	0 (0.0)	13 (0.1)	<0.001^*^^a^
Gastric erosion	2 (0.0)	1 (0.0)	0.385^a^	0 (0.0)	10 (0.0)	0.003^*^^a^
Senile tremor	10 (0.0)	0 (0.0)	<0.001^*^^a^	0 (0.0)	0 (0.0)	N/A
Medications No. (%)						
Central acetylcholinesterase inhibitor	11,086 (30.2)	15,556 (30.6)	0.238^a^	1,382 (7.2)	2093 (9.5)	<0.001^*^^a^
Second generation antipsychotic	5,505 (15.0)	8,490 (16.7)	<0.001^*^^a^	2,847 (14.8)	3,794 (17.2)	<0.001^*^^a^
Selective serotonin reuptake inhibitor	10,824 (29.5)	18,005 (35.4)	<0.001^*^^a^	4,798 (25.0)	7,194 (32.6)	<0.001^*^^a^
Memantine	7,051 (19.2)	10,408 (20.5)	<0.001^*^^a^	945 (4.9)	1,379 (6.3)	<0.001^*^^a^
Buspirone	2,581 (7.0)	5,556 (10.9)	<0.001^*^^a^	1,397 (7.3)	2,643 (12.0)	<0.001^*^^a^
Valproate	5,792 (15.8)	8,024 (15.8)	0.971^a^	2,650 (13.8)	3,073 (13.9)	0.710^a^
Lab values: Mean ± SD						
Folate (B9)	11.663 ± 4.0024	12.113 ± 4.1022	0.004^*^^b^	11.167 ± 4.1535	11.546 ± 4.3092	0.033^*^^b^
Cobalamin (B12)	516.50 ± 188.798	No data	N/A	1,023 ± 350.629	239	0.192^b^
Vitamin D	28.958 ± 13.2808	30.147 ± 16.0384	0.151^b^	27.027 ± 16.6856	27.802 ± 15.6052	0.529^b^
TSH	2.55875 ± 5.848485	3.32532 ± 11.630839	<0.001^*^^b^	2.92684 ± 7.870089	4.00290 ± 13.590204	<0.001^*^^b^
Magnesium	1.979 ± 0.3123	1.946 ± 0.3342	<0.001^*^^b^	1.951 ± 0.3481	1.915 ± 0.3480	<0.001^*^^b^
Serum calcium	9.102 ± 0.6404	9.157 ± 0.6818	<0.001^*^^b^	8.959 ± 0.7659	9.003 ± 0.7459	<0.001^*^^b^
Homocysteine	13.206 ± 5.5780	15.679 ± 18.7499	0.444^b^	16.346 ± 10.1423	13.045 ± 5.4778	0.071^b^

a Pearson’s Chi-Squared test.

b Student’s *T* test.

^*^ *p*-value <0.05. Results for continuous variables are presented as Mean ± SD, while discrete data are presented as percentage frequency. Pearson’s Chi-Square is used to compare differences between demographic and clinical characteristics in groups with being male or female in the presence of encephalopathy.

Female ADEN patients were generally older (*p* < 0.001), and presented with a range of medical histories, including insomnia, hypertension, dyslipidemia, osteoporosis, unspecified cancer, anxiety, urinary tract infections, upper respiratory infections, insulin use, GI ulceration, squamous cell lung carcinoma, unspecified headaches, congestive heart failure, OSA, cutaneous ulcers, chronic obstructive pulmonary disease, rheumatoid arthritis, and Down syndrome (*p* < 0.001). Furthermore, they exhibited gait dysfunction (*p* = 0.008), secondary dementia (*p* = 0.002), frailty (*p* = 0.004), vasospasm (*p* = 0.005), and gastric erosion (*p* = 0.003) more frequently than males. Females were also more likely to be prescribed atypical antipsychotics, SSRIs, memantine, and buspirone (*p* < 0.001). Regarding laboratory values, they were associated with higher serum folate (*p* = 0.033), thyroid-stimulating hormone (TSH) (*p* < 0.001), and calcium levels (*p* < 0.001).

Male AD patients without encephalopathy were more likely to have a history of alcohol and tobacco use (*p* < 0.001). They also showed a greater incidence of various medical conditions, including dyslipidemia, gait dysfunction, PVD, unspecified cancer, squamous cell lung carcinoma, OSA, cutaneous ulcers, psychosis, pneumonia, Down syndrome, and senile tremor (*p* < 0.001). Additionally, these patients exhibited gait dysfunction (*p* = 0.016), atrial fibrillation (*p* = 0.045), and mild cognitive impairment (*p* = 0.008). Furthermore, they were found to have a higher average serum magnesium level (*p* < 0.001).

Female patients were more likely to report a medical history that included insomnia, hypertension (HTN), osteoporosis, anxiety, urinary tract infections (UTI), upper respiratory infections (URI), unspecified thyroid disease, insulin use, secondary dementia, cold sores, GI ulceration, lung adenocarcinoma, small cell lung carcinoma, unspecified headaches, congestive heart failure (CHF), and rheumatoid arthritis (*p* < 0.001). They also had a higher occurrence of hallucinations (*p* = 0.016). On average, females more frequently used SGAs and SSRIs. Additionally, they were more likely to present with elevated levels of serum folate (*p* = 0.004), thyroid-stimulating hormone (TSH) (*p* < 0.001), and calcium (*p* < 0.001).

### Sex-stratified analysis and multivariate regression analysis

Table 3 highlights risk factors for AD patients with encephalopathy compared to those without encephalopathy. ADEN patients were more likely to be male (OR = 1.106, 95% CI, 1.076–1.137, *p* < 0.001), and present with PVD (OR = 1.232, 95% CI, 1.170–1.296, *p* < 0.001), atrial fibrillation (OR = 1.142, 95% CI, 1.089–1.197, *p* < 0.001), anxiety (OR = 1.121, 95% CI, 1.069–1.175, *p* < 0.033), urinary tract infections (UTI) (OR = 1.682, 95% CI, 1.616–1.751, *p* < 0.001), and take insulin (OR = 1.625, 95% CI, 1.431–1.845, *p* < 0.001). They were also more likely to present GI ulceration (OR = 1.529, 95% CI, 1.327–1.762, *p* < 0.001), small cell lung carcinoma (OR = 1.924, 95% CI, 1.413–2.619, *p* < 0.001), and squamous cell lung carcinoma (OR = 1.625, 95% CI, 1.046–2.524, *p* = 0.031). Furthermore, they had higher odds of presenting with congestive heart failure (OR = 1.732, 95% CI, 1.647–1.821, *p* < 0.001), chronic obstructive pulmonary disease (COPD) (OR = 1.194, 95% CI, 1.144–1.245, *p* < 0.001), pneumonia (OR = 2.132, 95% CI, 2.053–2.214, *p* < 0.001), rheumatoid arthritis (OR = 1.369, 95% CI, 1.226–1.529, *p* < 0.001), and a history of tobacco use (OR = 1.653, 95% CI, 1.607–1.699, *p* < 0.001). They were more likely to be treated with buspirone (OR = 1.126, 95% CI, 1.077–1.176, *p* < 0.001) and valproate (OR = 1.050, 95% CI, 1.012–1.090, *p* = 0.010).

**Table 3:** The adjusted analysis results for AD patients with encephalopathy and those without encephalopathy.

Variables	B value	Wald	Odds ratio	95% C.I.		*p*-value
				Lower	Upper	
Male gender	0.101	51.963	1.106	1.076	1.137	<0.001*
Hispanic ethnicity	−0.238	22.666	0.788	0.714	0.869	<0.001*
Insomnia	−0.360	165.018	0.698	0.661	0.737	<0.001*
Hypertension	−0.043	9.260	0.958	0.932	0.985	0.002*
Dyslipidemia	−0.231	196.704	0.794	0.769	0.820	<0.001*
Cerebrovascular accident	−0.070	9.866	0.932	0.892	0.974	0.002*
Osteoporosis	−0.266	115.879	0.766	0.730	0.804	<0.001*
Gait dysfunction	−0.516	154.472	0.597	0.550	0.648	<0.001*
Peripheral vascular disease	0.208	63.806	1.232	1.170	1.296	<0.001*
Atrial fibrillation	0.114	22.309	1.142	1.089	1.197	<0.001*
Anxiety	0.047	4.552	1.121	1.069	1.175	0.033*
Urinary tract infection	0.520	644.821	1.682	1.616	1.751	<0.001*
Upper respiratory infection	−0.535	117.025	0.586	0.532	0.646	<0.001*
Thyroid disease (unspecified)	−0.724	47.043	0.485	0.394	0.596	<0.001*
Insulin use	0.485	56.292	1.625	1.431	1.845	<0.001*
Secondary dementia	−1.153	277.181	0.316	0.276	0.362	<0.001*
Cold sore	−1.334	51.067	0.263	0.183	0.380	<0.001*
Frailty	−0.438	4.559	0.645	0.432	0.965	0.033*
Gastrointestinal ulceration	0.425	34.348	1.529	1.327	1.762	<0.001*
Small cell carcinoma of lung	0.654	17.305	1.924	1.413	2.619	<0.001*
Squamous cell carcinoma of lung	0.485	4.659	1.625	1.046	2.524	0.031*
Mild cognitive impairment	−2.053	1,429.205	0.128	0.115	0.143	<0.001*
Headache (unspecified)	−0.455	78.910	0.635	0.574	0.702	<0.001*
Congestive heart failure	0.549	460.633	1.732	1.647	1.821	<0.001*
Obstructive sleep apnea	−0.098	18.985	0.907	0.867	0.947	<0.001*
Psychosis	−0.448	29.275	0.639	0.544	0.752	<0.001*
Chronic obstructive pulmonary disease	0.177	68.138	1.194	1.144	1.245	<0.001*
Pneumonia	0.757	1,547.939	2.132	2.053	2.214	<0.001*
Rheumatoid arthritis	0.314	31.083	1.369	1.226	1.529	<0.001*
Down syndrome	−1.349	19.441	0.259	0.142	0.473	<0.001*
Alcohol consumption	−0.069	21.783	0.933	0.906	0.960	<0.001*
Tobacco use	0.502	1,244.330	1.653	1.607	1.699	<0.001*
Central acetylcholinesterase inhibitor	−1.323	3,816.223	0.266	0.255	0.278	<0.001*
Selective serotonin reuptake inhibitor	−0.131	82.811	0.877	0.852	0.902	<0.001*
Memantine	−0.795	933.441	0.452	0.429	0.475	<0.001*
Buspirone	0.118	27.693	1.126	1.077	1.176	<0.001*
Valproate	0.049	6.610	1.050	1.012	1.090	0.010*

Adjusted OR<1 denotes factors associated with AD without encephalopathy, while OR>1 denotes factors related to AD patients with encephalopathy. Hosmer-Lemeshow test (*p < 0.001*), Cox & Snell (*R*^2^ = 0.151). The overall classified percentage of 70.6% and area under the ROC curve (AUC = 0.742, 0.739–0.745) were applied to check model fitness. * Indicates statistical significance (*p* < 0.05) with a 95% confidence interval. Backward Stepwise model based on Likelihood Ratio was applied. Model assumptions were fulfilled. Multicollinearity and interactions among independent variables were checked and no significant interactions were found. Hosmer-Lemeshow test (*p* < 0.001), Cox & Snell (*R*^2^ = 0.151).

AD patients without encephalopathy were more likely to be Hispanic (OR = 0.788, 95% CI, 0.714–0.869, *p* < 0.001) and present with a history of insomnia (OR = 0.698, 95% CI, 0.661–0.737, *p* < 0.001), hypertension (OR = 0.958, 95% CI, 0.932–0.985, *p* = 0.002), dyslipidemia (OR = 0.794, 95% CI, 0.769–0.820, *p* < 0.001), cerebrovascular accidents (OR = 0.932, 95% CI, 0.892–0.974, *p* = 0.002), osteoporosis (OR = 0.766, 95% CI, 0.730–0.804, *p* < 0.001), and gait dysfunction (OR = 0.597, 95% CI, 0.550–0.648, *p* < 0.001). Other associated conditions included upper respiratory infections (URI) (OR = 0.586, 95% CI, 0.532–0.646, *p* < 0.001), unspecified thyroid disease (OR = 0.485, 95% CI, 0.394–0.596, *p* < 0.001), secondary dementia (OR = 0.316, 95% CI, 0.276–0.362, *p* < 0.001), cold sores (OR = 0.263, 95% CI, 0.183–0.380, *p* < 0.001), frailty (OR = 0.645, 95% CI, 0.432–0.965, *p* = 0.033), mild cognitive impairment (OR = 0.128, 95% CI, 0.115–0.143, *p* < 0.001), and unspecified headaches (OR = 0.635, 95% CI, 0.574–0.702, *p* < 0.001). Other notable factors included OSA (OR = 0.907, 95% CI, 0.867–0.947, *p* < 0.001), psychosis (OR = 0.639, 95% CI, 0.544–0.752, *p* < 0.001), Down syndrome (OR = 0.259, 95% CI, 0.142–0.473, *p* < 0.001), and alcohol use (OR = 0.933, 95% CI, 0.906–0.960, *p* < 0.001). AD patients without encephalopathy were more likely to be treated with ChEIs was associated with an odds ratio of (OR = 0.266, 95% CI, 0.255–0.278, *p* < 0.001), (SSRIs) (OR = 0.877,95% CI, 0.852–0.902, *p* < 0.001), and memantine (OR = 0.452, 95% CI, 0.429–0.475, *p* < 0.001). The logistic regression model demonstrated a strong predictive power with an area under the curve (AUC) of 0.742 (95% CI, 0.739–0.745, *p* < 0.001).

Table 4 highlights the stronger association between male sex and alcohol (OR = 1.555, 95% CI, 1.504–1.607, *p* < 0.001), tobacco use (OR = 2.591, 95% CI, 2.514–2.670, *p* < 0.001), dyslipidemia (OR = 1.234, 95% CI, 1.192–1.278, *p* < 0.001), gait dysfunction (OR = 1.169, 95% CI, 1.086–1.258, *p* < 0.001), PVD (OR = 1.435, 95% CI, 1.349–1.527, *p* < 0.001), unspecified cancer (OR = 2.573, 95% CI, 2.045–3.238, *p* < 0.001), frailty (OR = 1.977, 95% CI, 1.402–2.788, *p* < 0.001), mild cognitive impairment (OR = 1.090, 95% CI, 1.031–1.153, *p* = 0.003), OSA (OR = 1.460, 95% CI, 1.392–1.533, *p* < 0.001), cutaneous ulcer (OR = 2.426, 95% CI, 1.588–3.706, *p* < 0.001), psychosis (OR = 1.383, 95% CI, 1.175–1.629, *p* < 0.001), pneumonia (OR = 1.198, 95% CI, 1.138–1.260, *p* < 0.001), Down syndrome (OR = 3.644, 95% CI, 2.507–5.298, *p* < 0.001), and the use of memantine (OR = 1.076, 95% CI, 1.037–1.117, *p* < 0.001).

**Table 4:** The adjusted analysis results for male and female AD patients without encephalopathy.

Variables	B value	Wald	Odds ratio	95% C.I.		*p*-value
				Lower	Upper	
Alcohol	0.441	686.901	1.555	1.504	1.607	<0.001*
Tobacco	0.952	3856.611	2.591	2.514	2.670	<0.001*
Insomnia	−0.160	30.444	0.852	0.805	0.902	<0.001*
Dyslipidemia	0.210	141.198	1.234	1.192	1.278	<0.001*
Osteoporosis	−1.282	1862.300	0.278	0.262	0.294	<0.001*
Gait dysfunction	0.156	17.185	1.169	1.086	1.258	<0.001*
Peripheral vascular disease	0.361	130.914	1.435	1.349	1.527	<0.001*
Cancer (unspecified)	0.945	65.011	2.573	2.045	3.238	<0.001*
Anxiety	−0.443	288.310	0.642	0.610	0.676	<0.001*
Urinary tract infection	−0.729	705.460	0.482	0.457	0.509	<0.001*
Upper respiratory infection	−0.368	52.180	0.692	0.626	0.765	<0.001*
Thyroid disease (unspecified)	−0.548	27.456	0.578	0.471	0.710	<0.001*
Insulin use	−0.573	38.685	0.564	0.470	0.675	<0.001*
Cold sore	−1.234	62.139	0.291	0.214	0.396	<0.001*
Frailty	0.682	15.092	1.977	1.402	2.788	<0.001*
Gastrointestinal ulceration	−1.628	134.870	0.196	0.149	0.258	<0.001*
Lung adenocarcinoma	−2.083	92.140	0.125	0.081	0.191	<0.001*
Small cell carcinoma of lung	−2.544	68.446	0.079	0.043	0.143	<0.001*
Mild cognitive impairment	0.086	9.102	1.090	1.031	1.153	0.003*
Headache (unspecified)	−1.663	482.037	0.190	0.163	0.220	<0.001*
Congestive heart failure	−0.146	18.652	0.864	0.809	0.923	<0.001*
Obstructive sleep apnea	0.379	235.962	1.460	1.392	1.533	<0.001*
Cutaneous ulcer	0.886	16.801	2.426	1.588	3.706	<0.001*
Psychosis	0.324	15.160	1.383	1.175	1.629	<0.001*
Pneumonia	0.181	48.410	1.198	1.138	1.260	<0.001*
Rheumatoid arthritis	−0.716	74.369	0.489	0.415	0.575	<0.001*
Down syndrome	1.293	45.893	3.644	2.507	5.298	<0.001*
Selective serotonin reuptake inhibitor	−0.197	148.571	0.821	0.795	0.847	<0.001*
Memantine	0.073	14.823	1.076	1.037	1.117	<0.001*
Buspirone	−0.412	227.726	0.662	0.627	0.698	<0.001*

Adjusted OR<1 denotes factors associated with females while OR>1 denotes factors associated with male patients. Hosmer-Lemeshow test (*p* < 0.001), Cox & Snell (*R*^2^ = 0.133). The overall classified percentage of 66.2% and area under the ROC curve (AUC = 0.712, 0.709–0.716) were applied to check model fitness. * Indicates statistical significance (*p* < 0.05) with a 95% confidence interval. Backward Stepwise model based on Likelihood Ratio was applied. Model assumptions were fulfilled. Multicollinearity and interactions among independent variables were checked and no significant interactions were found. Hosmer-Lemeshow test (*p* < 0.001), Cox & Snell (*R*^2^ = 0.133).

Female AD patients without encephalopathy were associated with insomnia (OR = 0.852, 95% CI, 0.805–0.902, *p* < 0.001), osteoporosis (OR = 0.278, 95% CI, 0.262–0.294, *p* < 0.001), and anxiety (OR = 0.642, 95% CI, 0.610–0.676, *p* < 0.001). Additionally, they showed an association with urinary tract infections (UTI) (OR = 0.482, 95% CI, 0.457–0.509, *p* < 0.001), upper respiratory infections (URI) (OR = 0.692, 95% CI, 0.626–0.765, *p* < 0.001), and unspecified thyroid disease (OR = 0.578, 95% CI, 0.471–0.710, *p* < 0.001). Moreover, females were more likely to take insulin (OR = 0.564, 95% CI, 0.470–0.675, *p* < 0.001), present with cold sores (OR = 0.291, 95% CI, 0.214–0.396, *p* < 0.001), GI ulceration (OR = 0.196, 95% CI, 0.149–0.258, *p* < 0.001),lung adenocarcinoma (OR = 0.125, 95% CI, 0.081–0.191, *p* < 0.001), small cell lung carcinoma (OR = 0.079, 95% CI, 0.043–0.143, *p* < 0.001), unspecified headaches (OR = 0.190, 95% CI, 0.163–0.220, *p* < 0.001), and congestive heart failure (OR = 0.864, 95% CI, 0.809–0.923, *p* < 0.001). Furthermore, they were more likely to present with rheumatoid arthritis (OR = 0.489, 95% CI, 0.415–0.575, *p* < 0.001), and treated with SSRIs (OR = 0.821, 95% CI, 0.795–0.847, *p* < 0.001) and buspirone (OR = 0.662, 95% CI, 0.627–0.698, *p* < 0.001). The logistic regression analysis model has robust predictive power (AUC = 0.712, 95% CI, 0.709–0.716, *p* < 0.001).

Table 5 highlights the stronger association between male sex and a history of alcohol (OR = 2.026, 95% CI, 1.931–2.125, *p* < 0.001) and tobacco use (OR = 2.459, 95% CI, 2.346–2.577, *p* < 0.001). Males presented with higher odds of presenting with hypertension (OR = 1.144, 95% CI, 1.094–1.197, *p* < 0.001), PVD (OR = 1.606, 95% CI, 1.485–1.737, *p* < 0.001), atrial fibrillation (OR = 1.555, 95% CI, 1.443–1.676, *p* < 0.001), hallucinations (OR = 1.406, 95% CI, 1.119–1.766, *p* = 0.003), and traumatic head injury (OR = 3.211, 95% CI, 2.346–4.395, *p* < 0.001). Additionally, they were more likely to be treated with memantine (OR = 1.133, 95% CI, 1.032–1.243, *p* = 0.009).

**Table 5:** The results for the adjusted analysis for male and female ADEN patients.

Variables	B value	Wald	Odds ratio	95% C.I.		*p*-value
				Lower	Upper	
Hispanic ethnicity	0.265	8.997	1.304	1.096	1.551	0.003*
Alcohol	0.706	840.600	2.026	1.931	2.125	<0.001*
Tobacco	0.900	1,408.098	2.459	2.346	2.577	<0.001*
Hypertension	0.135	34.423	1.144	1.094	1.197	<0.001*
Osteoporosis	−1.180	531.094	0.307	0.278	0.340	<0.001*
Peripheral vascular disease	0.474	141.382	1.606	1.485	1.737	<0.001*
Atrial fibrillation	0.442	134.033	1.555	1.443	1.676	<0.001*
Hallucination	0.341	8.577	1.406	1.119	1.766	0.003*
Cancer (unspecified)	−0.486	26.753	0.615	0.512	0.740	<0.001*
Anxiety	−0.497	174.279	0.609	0.565	0.655	<0.001*
Urinary tract infection	−0.796	581.931	0.451	0.423	0.481	<0.001*
Upper respiratory infection	−0.633	35.903	0.531	0.432	0.653	<0.001*
Insulin use	−0.477	22.196	0.620	0.509	0.757	<0.001*
Gastrointestinal ulceration	−1.086	87.133	0.338	0.269	0.424	<0.001*
Squamous cell carcinoma of lung	−2.598	12.548	0.074	0.018	0.313	<0.001*
Headache (unspecified)	−0.695	48.879	0.499	0.411	0.606	<0.001*
Congestive heart failure	−0.226	35.802	0.798	0.741	0.859	<0.001*
Cutaneous ulcer	−0.949	6.968	0.387	0.191	0.783	0.008*
Chronic obstructive pulmonary disease	−0.268	69.353	0.765	0.718	0.814	<0.001*
Traumatic head injury	1.167	53.081	3.211	2.346	4.395	<0.001*
Rheumatoid arthritis	−1.194	139.314	0.303	0.249	0.370	<0.001*
Second generation antipsychotic	−0.061	4.040	0.941	0.887	0.998	0.044*
Selective serotonin reuptake inhibitor	−0.275	131.421	0.759	0.725	0.796	<0.001*
Memantine	0.125	6.883	1.133	1.032	1.243	0.009*
Buspirone	−0.571	223.375	0.565	0.524	0.609	<0.001*

Adjusted OR<1 denotes factors associated with females, while OR>1 denotes factors associated with male patients. Hosmer-Lemeshow test (*p* < 0.001), Cox & Snell (*R*^2^ = 0.137). The overall classified percentage of 66.3% and area under the ROC curve (AUC = 0.712, 0.707–0.717) were applied to check model fitness. * Indicates statistical significance (*p* < 0.05) with a 95% confidence interval. Backward Stepwise model based on Likelihood Ratio was applied. Model assumptions were fulfilled. Multicollinearity and interactions among independent variables were checked and no significant interactions were found. Hosmer-Lemeshow test (*p* < 0.001), Cox & Snell (*R*^2^ = 0.137).

Females were more likely to present with osteoporosis (OR = 0.307, 95% CI, 0.278–0.340, *p* < 0.001), unspecified cancer (OR = 0.615, 95% CI, 0.512–0.740, *p* < 0.001), anxiety (OR = 0.609, 95% CI, 0.565–0.655, *p* < 0.001), urinary tract infections (UTI) (OR = 0.451, 95% CI, 0.423–0.481, *p* < 0.001), and upper respiratory infections (URI) (OR = 0.531, 95% CI, 0.432–0.653, *p* < 0.001). They were also less likely to be taking insulin (OR = 0.620, 95% CI, 0.509–0.757, *p* < 0.001). Furthermore, females presented with GI ulceration (OR = 0.338, 95% CI, 0.269–0.424, *p* < 0.001), squamous cell lung carcinoma (OR = 0.074, 95% CI, 0.018–0.313, *p* < 0.001), unspecified headaches (OR = 0.499, 95% CI, 0.411–0.606, *p* < 0.001), congestive heart failure (CHF) (OR = 0.798, 95% CI, 0.741–0.859, *p* < 0.001), cutaneous ulcers (OR = 0.387, 95% CI, 0.191–0.783, *p* = 0.008), chronic obstructive pulmonary disease (COPD) (OR = 0.765, 95% CI, 0.718–0.814, *p* < 0.001), and rheumatoid arthritis (OR = 0.303, 95% CI, 0.249–0.370, *p* < 0.001). They were also more likely to be prescribed SGAs (OR = 0.941, 95% CI, 0.887–0.998, *p* = 0.044), SSRIs (OR = 0.759, 95% CI, 0.725–0.796, *p* < 0.001), and buspirone (OR = 0.565, 95% CI, 0.524–0.609, *p* < 0.001). The logistic regression model demonstrated was strong with a predictive power (AUC = 0.709, 95% CI, 0.707–0.717, *p* < 0.001) (Table 6).

**Table 6:** The summary for differences and similarities in male and female patients with ADEN.

AD w/o encephalopathy		ADEN	
Males	Females	Males	Females
Alcohol use	Insomnia	Alcohol use	Unspecified cancer
Tobacco use	Osteoporosis	Tobacco use	Urinary tract infections
Dyslipidemia	Anxiety	Hypertension	Osteoporosis
Gait dysfunction	Urinary tract infection	Atrial fibrillation	Upper respiratory infections
Peripheral vascular disease	Upper respiratory infections	Hallucinations	Anxiety
Unspecified cancer	Unspecified thyroid disease	Traumatic head injury Memantine	Insulin
Frailty	Insulin use		Gastrointestinal Ulceration
Mild cognitive impairment	Cold sores		Squamous cell lung carcinoma
Cutaneous ulcer	Gastrointestinal ulceration		Headaches
Psychosis	Lung adenocarcinoma		Congestive heart failure
Pneumonia	Small cell lung carcinoma		Cutaneous ulcers
Down syndrome	Headaches		Chronic obstructive pulmonary disease Rheumatoid arthritis Selective Serotonin Reuptake Inhibitors Second generation antipsychotics Buspirone
Memantine	Congestive heart failure		
	Selective serotonin reuptake inhibitors		
	Rheumatoid arthritis		
	Buspirone		

## Discussion

As the prevalence of ADEN continues to rise, researchers need to address the various risk factors underpinning its epidemiology. It is often noted that sex differences have been overlooked in this critical discourse (Wang, 2024). This study highlights the significant influences of risk factors contributing to sex differences in patients diagnosed with ADEN. Our findings reveal different risk factors that contribute to sex differences in ADEN patients. We observed that, at 95% CI, male patients are more likely to be Hispanic, have a history of alcohol and tobacco use, hypertension, PVD, atrial fibrillation, hallucination, traumatic head injury, and are treated with memantine. In contrast, our analysis revealed that at 95% CI, female patients are likely to present with osteoporosis, unspecified cancer, anxiety, and urinary tract infections and are placed on insulin use.

Improper use of insulin can lead to hypoglycemia or low blood sugar (Dave et al., 2023). This condition may result in hypoglycemic encephalopathy or metabolic encephalopathy (Dave et al., 2023). Additionally, insulin resistance and high levels of insulin (hyperinsulinemia) have been associated with an increased risk of certain types of cancer, including breast, colon, and pancreatic cancers (Szablewski, 2024). This association is frequently discussed regarding Type 2 diabetes, where insulin resistance is a significant characteristic (Szablewski, 2024). While insulin may encourage cell proliferation and tumor growth (Leitner et al., 2022), the specific mechanisms involved and the extent of the associated risk are still being investigated and warrant further research in future studies.

In addition, male patients are more likely to present with GI ulcers, squamous cell lung carcinoma, headache, CHF, cutaneous ulcer, COPD, and rheumatoid arthritis. Moreover, Females are more likely to be treated with SGAs, SSRIs, and buspirone.

We observed that alcohol and tobacco use were associated with male ADEN patients. This result was significant at 95% CI, tobacco and alcohol use are related as smokers drink and drinkers smoke (US Department of Health and Human Services, 1998; Bailey-Taylor et al., 2022). The heaviest drinkers are the most likely to smoke, and 70 to almost 100% of alcoholics in treatment programs report smoking (Bobo and Husten, 2000). The observation that alcohol and tobacco use appear to influence each other’s association with AD is consistent with evidence of a biological interaction between smoking and drinking (Ning et al., 2020). This observation also may be attributed, however, to the increased overall mortality of people who both smoke and drink, a possibility that can only be ruled out by longitudinal research (Hongli et al., 2021). Our finding of alcohol and tobacco use being associated with ADEN is consistent with evidence that both alcohol and tobacco use are significantly associated with an increased risk of AD in male patients (Seemiller et al., 2024; Raulin et al., 2022). These studies show that heavy consumption of either substance can potentially accelerate the progression of the disease and increase the likelihood of developing AD, particularly when combined with other risk factors like genetics. Therefore, alcohol and tobacco use may have significant effects on ADEN, potentially worsening their condition or complicating their management. The specific relationship between Hispanic males who smoke and drink with ADEN is unknown and should be investigated in future longitudinal research.

The presence of hypertension, PVD, and atrial fibrillation often co-occur and collectively increase the risk of cardiovascular events and complications due to their shared risk factors like age, diabetes, and smoking, further impacting cognitive function (Petrie et al., 2018) and overall health in AD (Justin et al., 2013; Iadecola et al., 2016; Badji et al., 2023) and encephalopathy patients (Tapper et al., 2019; Jin et al., 2024). While each condition can independently increase the risk of stroke in AD or encephalopathy patients, our finding that hypertension, PVD, and atrial fibrillation are associated with male ADEN patients at 95% CI reveal this concerning combination in increasing the likelihood of a cerebrovascular event due to their shared underlying pathology of atherosclerosis and impaired blood flow to the brain. Therefore, careful monitoring and management of these conditions is crucial in ADEN patients.

In AD patients, hallucinations result in sensory perceptions, including seeing, hearing, or feeling things that do not exist (Burghaus et al., 2012), and this can occur in the advanced stages of AD, frequently manifesting as visual hallucinations of people or objects that are not real (El Haj et al., 2017). These hallucinations can be distressing and require careful management by a caregiver. In encephalopathy, hallucination could originate from liver cirrhosis (hepatic encephalopathy), where toxins build up in the blood, leading to altered mental states, including confusion and hallucinations (Nourbakhsh and Ferrando, 2024); auditory hallucinations are less common in encephalopathy patients (Al-Dury et al., 2021). We observed that there is an association between hallucinations and male ADEN patients. This finding suggests the need to develop hallucination-focused integrative therapy that targets solely due to their prevalence as one of the most frequent psychotic symptoms in AD and or encephalopathy patients.

A single head injury can increase the risk of developing dementia, which increases with repeated head traumas, which can lead to a condition known as Chronic Traumatic Encephalopathy (CTE) (Shively et al., 2012). At 95% CI, we observed an association of traumatic head injury in male ADEN patients. This result is not surprising as traumatic head injury can significantly increase the risk of developing AD and encephalopathy which can significantly worsen cognitive functions, potentially accelerating the progression of their neurodegenerative disease in male and female patients (Brett et al., 2022).

We observed that memantine was associated with male ADEN patients. Memantine is an SSRI used to treat AD patients. Higher doses of memantine in AD patients are associated with kidney disorders (Prasad et al., 2022). While our current results do not support the possibility that memantine directly “causes” AD or encephalopathy in male patients, further studies are necessary to understand the predisposition of ADEN patients who present with chronic kidney disease to develop adverse effects related to memantine accumulation.

At 95% CI, our analysis reveals that female ADEN present with osteoporosis, unspecified cancer, anxiety, and urinary tract infections and are placed on insulin use. In addition, they are more likely to present with GI ulcers, squamous cell lung carcinoma, headache, CHF, cutaneous ulcer, COPD, and rheumatoid arthritis. Moreover, Females are more likely to be treated with SGAs, SSRIs, and buspirone. Both SGAs and SSRIs present with a variety of side effects. SGAs may lead to metabolic concerns such as weight gain and diabetes, while SSRIs can cause gastrointestinal issues like diarrhea and nausea. Furthermore, both classes of medications may result in sexual dysfunction and, in certain instances, provoke suicidal thoughts, particularly among young adults.

Our observations indicate that female patients with ADEN are at a greater risk of being diagnosed with osteoporosis. As AD progresses, patients often experience notable changes in body composition, which further links AD with osteoporosis and an increased likelihood of fractures (Lary et al., 2021). The coexistence of these conditions may lead to even more severe health outcomes. As a result, female ADEN patients not only face a higher risk of fractures but may also experience an increased risk of mortality in the event of a fracture. Thus, there is an urgent need for future large-scale studies to investigate further the relationship between osteoporosis and dementia, especially among female ADEN patients.

Cancer and AD demonstrate complex interrelationships marked by shared risk factors, overlapping biological pathways, and treatment implications (Zabłocka et al., 2021). This association points to a potential connection between cancer, encephalopathy, and AD, particularly regarding their underlying pathogenesis (Shafi, 2016). The relationship between cancer and female patients with ADEN underscores the importance of developing both observational and active surveillance strategies. This proactive approach would help reduce the healthcare burden on ADEN patients by enabling early intervention and managing their intricate health conditions.

In the current study, female patients with ADEN were associated with anxiety. Anxiety is often linked to various forms of encephalopathy, including those resulting from encephalitis or liver disease (Butterworth, 2003). Furthermore, the cognitive and emotional changes associated with encephalopathy can contribute to heightened anxiety levels (Howlett et al., 2022). The prevalence of anxiety symptoms among AD patients ranges from 9 to 70%, with anxiety disorders reported in approximately 5 to 20% of this population (Mendez, 2021). In individuals suffering from encephalopathy, the presence of anxiety exacerbates the already complex symptoms of dementia, leading to increased caregiver burden and stress (Boltz et al., 2015). Therefore, it is essential to establish specific diagnostic criteria and develop screening tools tailored for female ADEN patients to enhance clinical outcomes and alleviate the strain on caregivers.

Insulin, a hormone essential for regulating blood sugar levels, has been linked to AD and encephalopathy in various ways (Cholerton et al., 2013). For instance, while insulin therapy is critical for managing diabetes, it can occasionally lead to a condition known as hypoglycemic encephalopathy (McCall, 2012). Furthermore, insulin plays a multifaceted role in AD. Insulin resistance, diabetes, and low insulin levels are all correlated with an increased risk of cognitive decline (Kim and Feldman, 2015). Therefore, maintaining proper blood sugar control and enhancing insulin sensitivity may offer protection against ADEN, particularly in female patients.

Female patients with ADEN were associated with urinary tract infections (UTIs). If left untreated, UTIs, particularly those caused by urease-producing bacteria, can result in hyperammonemia encephalopathy, a condition characterized by elevated ammonia levels in the blood that can damage the brain (Gorantla et al., 2022). Additionally, UTIs are especially prevalent among patients with AD due to age-related risk factors such as malnutrition (Carlsson et al., 2013), poorly managed diabetes (Nitzan et al., 2015), and inadequate bladder control (Rowe and Juthani-Mehta, 2013), which can lead to urinary retention or incontinence (Salari et al., 2022). The diverse presentations of these infections in female ADEN patients highlight the critical importance of timely detection and treatment to prevent further cognitive decline and improve overall health outcomes.

In the current study, female patients with ADEN were associated with rheumatoid arthritis (RA). RA mainly affects the joints and can also result in extra-articular manifestations, including potential neurological complications (Cojocaru et al., 2010). While RA primarily affects joints, it can also cause encephalopathy. Some cases of autoimmune encephalitis have been linked to RA (Kitamura et al., 2019), highlighting the potential for neurological complications in RA patients. Several studies have explored the relationship between RA and AD (Sangha et al., 2020; Policicchio et al., 2017), and findings indicate that chronic inflammation, oxidative stress, and vascular disease associated with RA may elevate the risk of developing AD (Steven et al., 2019). Given that RA is linked to chronic systemic inflammation and is more prevalent in females (Guo et al., 2018), it serves as a clinical risk factor for investigating the biological mechanisms of inflammation and its possible role in cognitive decline among female patients with ADEN.

The current study found an association between gastrointestinal (GI) ulcers and female patients with ADEN. While GI ulcers do not directly cause encephalopathy (Uruha et al., 2011), severe complications such as bleeding and malabsorption can indirectly lead to Wernicke’s encephalopathy due to thiamine deficiency or hyperammonemic encephalopathy (Huertas-González et al., 2015). Individuals with GI ulcers may experience a higher incidence of AD compared to those without GI ulcers (Choi et al., 2020). Our findings highlight the relationship between GI ulcers, encephalopathy, and dementia in female patients, underscoring the complex nature of ADEN. Future research should explore the mechanisms through which specific GI ulcers may impact female ADEN patients. Such studies present an opportunity for a more comprehensive understanding of ADEN, which could inform prevention and treatment strategies for affected individuals.

We observed that squamous cell lung carcinoma was associated with female ADEN patients. Squamous cell lung carcinoma is related to encephalopathy, and this may manifest as paraneoplastic limbic encephalitis or other neurological complications (Sauri et al., 2015). Although lung cancer and AD are distinct conditions, there is a possibility that lung cancer patients, particularly those undergoing treatment, may have an increased risk of cognitive impairment and potentially AD, possibly due to treatment side effects or underlying biological factors (Kao et al., 2023; Simó et al., 2015). Further research is necessary to fully elucidate the relationship between squamous cell lung carcinoma, encephalopathy, and AD, particularly regarding the specific mechanisms at play and the influence of different cancer types, including squamous cell lung carcinoma in female patients with ADEN.

Female ADEN patients in our study were associated with headaches. Headaches linked to encephalopathy may indicate brain inflammation and are often associated with encephalitis and hypertensive encephalopathy (Hobson et al., 2012). While headaches and AD are distinct conditions, they can sometimes occur together. Evidence indicates a potential link between them; for instance, certain headache types, like migraines, may increase the risk of developing AD (Kim et al., 2023). While migraines and chronic daily headaches may be tied to a heightened risk of AD (Lee et al., 2021), but further research is necessary to explore the underlying mechanisms, particularly through longitudinal studies that assess cognitive function across different migraine phases, including migraine with aura and chronic migraine in patients with ADEN patients.

We observed an association between congestive heart failure (CHF) and female patients diagnosed with ADEN patients. CHF can result in neurologic dysfunction, including cardiac encephalopathy, which is characterized by confusion, lethargy, and cognitive or behavioral abnormalities (Caplan, 2004). These symptoms often arise from changes within the cranial cavity due to fluid retention and decreased cardiac output (Caplan, 2004). Additionally, CHF is associated with an increased risk of AD, as the condition can adversely affect the brain and lead to cognitive impairment (Goh et al., 2022). Our findings highlight the relationship between CHF and ADEN in female patients. Given these associations, large-scale prospective studies are essential for further investigating the interrelationships between CHF and the risk and incidence of ADEN among women.

We observed that female patients with ADEN are more likely to present with chronic obstructive pulmonary disease (COPD). Individuals with COPD may face neurological challenges, including cognitive impairment and the potential onset of posterior reversible encephalopathy syndrome, which manifests through symptoms such as headaches, seizures, and altered mental status (Regmi et al., 2022). COPD patients are at a heightened risk for developing AD compared to those without COPD (Awatade et al., 2023). This association persists even when accounting for the presence of vascular disease, underscoring COPD as an independent predictor of AD (Awatade et al., 2023). Consequently, it is crucial to ensure proper clinical management and focus on preventing or mitigating the incidence of COPD in female patients with AD.

We observed that female ADEN patients are more likely to be treated with SGAs and SSRIs, including buspirone. SSRIs are the most commonly prescribed medication for male and female AD patients, although females were prescribed them more often than males (Thunander Sundbom et al., 2017), supporting our current result. Moreover, SGAs are often used as an “augmentation” therapy, added to an SSRI medication when a patient is not responding well enough to the SSRI alone, particularly in cases of treatment-resistant depression (TRD); essentially, an SGA can be added to enhance the effectiveness of an SSRI for patients with severe depressive symptoms that cannot fully be managed by the SSRI alone. It is possible our female ADEN patients received a combined SGA and SSRI therapy to address depressive symptoms. Since the use of SSRIs may be associated with lower insulin secretion in nondiabetic participants and an increased risk of insulin dependence. The association of insulin use in our female patients may have served as an exogenous insulin use to achieve glycemic control and manage blood sugar levels.

### Clinical implications

Existing literature generally points to biological and environmental factors influencing sex differences and risk factors for various diseases (Keenan et al., 2025; Aggarwal and Mielke, 2023). Biological factors include sex chromosomes, hormones, and genetic variations affecting gene expression and disease susceptibility. Gender, encompassing social and cultural influences, also impacts lifestyle choices. Our current study reveals specific sex-related risk factors that can be managed to improve the care of male and female patients with ADEN. Future studies focusing on understanding the mechanisms behind sex differences in risk factors in ADEN can help develop more effective prevention and treatment strategies for both men and women, leading to improved health outcomes for ADEN patients.

## Conclusion

We observed differences in risk factors contributing to sex differences among ADEN patients. Since male and female ADEN patients present with differences in risk factors, effective therapeutic management of dementia patients should consider improved strategies in pharmacologic treatments to eliminate identified sex differences in the treatment of ADEN patients for males and females. Additionally, this study’s findings suggest that further investigation into disparities in risk factors encountered by male and female ADEN patients should be conducted by developing pertinent strategies. These findings underscore the need for sex-specific clinical approaches in managing ADEN. Future studies should explore whether interventions targeting identified risk factors such as cardiovascular health in males and immune/inflammatory conditions in females, can reduce ADEN incidence or severity. In addition, sex-specific clinical guidelines and screening tools acknowledge that men and women may experience diseases and respond to treatments differently. The development of such guidelines and tools for ADEN patients may provide more accurate and personalized care by considering biological sex.

### Limitations

This retrospective data analysis was from a single institution, so the data cannot be generalized to other institutions and populations. Additionally, electronic medical records (EMR) were used for the data analysis, limiting the patient information we could analyze. For example, there was no access to MiniMental State Examination (MMSE) data, medication therapy length, dosages, behavioral and psychological diagnoses, or a condition history to explain the length of hospital stay, whether it be different types of encephalopathy or related to another comorbidity. Along with these limitations, electronic medical records (EMRs) allow for the possibility of human error to limit the efficacy of the results. The database did not contain information about the diagnosis of encephalopathy. Race was classified as Black, White, and others. This makes further expansion of the other categorizations difficult. In addition to these limitations, this study uses terms to describe sex interchangeably, as the EMR data does not distinguish between them. The complexities of sex differences arise from various factors, including genetics, hormones, and environmental influences. Our findings highlight risk factors for both male and female ADEN patients within this context of differences.

## Data Availability

The datasets presented in this study can be found in online repositories. The names of the repository/repositories and accession number(s) can be found in the article/supplementary material.
